# miR-200-3p suppresses cell proliferation and reduces apoptosis in diabetic retinopathy via blocking the TGF-β2/Smad pathway

**DOI:** 10.1042/BSR20201545

**Published:** 2020-11-25

**Authors:** Liping Xue, Cheng Xiong, Juanjuan Li, Yuling Ren, Liwei Zhang, Kangwei Jiao, Chen Chen, Peng Ding

**Affiliations:** 1 Department of Ophthalmology, Yunnan Eye Institute, Yunnan No.2 Provincial People’s Hospital, Kunming 650021, Yunnan, China; 2 Department of Neurosurgery, The First Affiliated Hospital of Kunming Medical University, Kunming 650032, Yunnan, China

**Keywords:** diabetic retinopathy, high glucose, miR-200a-3p, TGF-β2/Smad pathway

## Abstract

Increasing evidence has shown that microRNAs (miRNAs) play an important role in the pathogenesis of diabetic retinopathy (DR). However, the role and mechanism of miRNA in regulating high glucose (HG)-induced ARPE-19 cell injury are still not well understood. The present study aimed to investigate the effects of miR-200a-3p on DR progression and reveal the underlying mechanisms of their effects. In the present study, we observed that miR-200a-3p was significantly decreased, while transforming growth factor-β2 (TGF-β2) expression was up-regulated in ARPE-19 cells treated with HG and retina tissues of DR rats. Subsequently, overexpression of miR-200a-3p significantly promoted cell proliferation, reduced apoptosis, as well as inhibited the levels of inflammatory cytokines secreted, matrix metalloprotease 2/9 (MMP2/9), and vascular endothelial growth factor (VEGF) in HG-injured ARPE-19 cells. Moreover, miR-200a-3p was proved to target TGF-β2 mRNA by binding to its 3′ untranslated region (3′UTR) using a luciferase reporter assay. Mechanistically, overexpression of miR-200a-3p reduced HG-induced ARPE-19 cell injury and reduced inflammatory cytokines secreted, as well as down-regulated the expression of VEGF via inactivation of the TGF-β2/Smad pathway *in vitro*. *In vivo* experiments, up-regulation of miR-200a-3p ameliorated retinal neovascularization and inflammation of DR rats. In conclusion, our findings demonstrated that miR-200a-3p-elevated prevented DR progression by blocking the TGF-β2/Smad pathway, providing a new therapeutic biomarker for DR treatment in the clinic.

## Introduction

Diabetic retinopathy (DR) is one of the most common and serious complications of diabetic mellitus [1]. The worldwide prevalence of DR from a pooled meta-analysis of population studies conducted in the United States, Australia, Europe, and Asia reported an overall prevalence of 35% for any type of DR [2]. In the coming years, the prevalence of DR will rapidly increase with the increasing number and lifespan of people with diabetes. It is caused by abnormal retinal blood vessels that are either proliferatively or functionally incompetent, leading fluid and lipid into the retina and causing visual impairment [3]. However, there are no treatment measures currently to effectively restrict DR progression and the mechanism of DR remains unknown.

microRNA (miRNA) is a family of short (20–24 nucleotides in length) single-stranded RNA, which binds to partially complementary sites primarily found in the 3′ untranslated region (3′UTR) of target mRNA and inhibits gene expression via induction of mRNA degradation and translational repression [4,5]. The regulatory functions of miRNAs were widespread, including cellular proliferation, apoptosis, differentiation, inflammatory cytokines secretion, tumorigenesis, organogenesis, hematopoiesis, and infection prevention to microbes including virus [6]. Previous studies indicated that miRNAs play an important role in regulating angiogenesis [7–9]. miR-200 family was closely associated with vascular endothelial cells and the functions of blood vessels [10]. It is a kind of angiogenesis signal-regulating factor, which is important for the maintenance of vascular endothelial cells and blood vessels’ integrity in the body. Furthermore, miR-200a [11], miR-200b [12], and miR-200c [13] were aberrantly expressed in the retinas of diabetic mellitus patients/rats, which indicated the potential role of the miR-200 family in the pathogenesis of DR. However, the influence of miR-200a-3p on DR progression is needed to be elucidated further.

The transforming growth factor-β/Smad (TGF-β/Smad) signaling pathway is involved in many cellular processes in mature organisms and developing embryos, including cell growth, differentiation, apoptosis, dynamic balance, and other cellular functions [14]. Besides, previous studies have confirmed that the activation of the TGF-β/Smad pathway plays an important role in regulating angiogenesis, extracellular matrix synthesis, and expression of inflammatory cytokines in the eye tissues [15,16]. TGF-β2 is one of the TGF-β family, which exerts biological functions by binding to heteromeric serine/threonine kinase transmembrane receptor complex consisting of type I and type II receptors [29293886]. TGF-β mediated the ligand binding of type II receptors and stimulation of type I receptor serine/threonine kinase activity via phosphorylated receptor-associated Smads proteins [17]. For example, Yafai et al. reported that the up-regulation of TGF-β2 inhibited the proliferation of retinal endothelial cells by enhancing the expression of Smad2 and Smad3 [18]. Recently, it is reported that inhibition of TGF-β2/Smad contributed to alleviating proinflammatory cytokines secretion [19] and decreasing endothelial angiogenic activities [20]. Importantly, overexpression of TGF-β2 is associated with the levels of matrix metalloproteinase-2 (MMP2), MMP9, and vascular endothelial growth factor (VEGF) in DR patients’ vitreous samples [21], indicating that blocking the TGF-β2/Smad pathway might be a beneficial therapeutic approach for DR.

In the present study, the expression level of miR-200a-3p and TGF-β2 in human retinal pigment epithelium cells (ARPE-19) treated with high glucose (HG) and the retinal tissues of DR rats, and the targeting relationship between miR-200a-3p and TGF-β2 were investigated, which could regulate the proliferation and apoptosis of HG pretreated ARPE-19 cells *in vitro*. Furthermore, the mechanism of miR-200a-3p mediated the progression of DR via regulating the TGF-β2/Smad pathway was substantiated *in vivo*. Taken together, our study will provide vital theoretical evidence for explaining the mechanisms of the miR-200a-3p/TGF-β2/Smad pathway in DR progression, and at the same time will provide a new biomarker and target for the diagnosis and treatment of DR.

## Materials and methods

### Cell culture and transfection

ARPE-19 cells were purchased from the Shanghai Institutes for Biological Sciences of the Chinese Academy of Sciences. These cells were cultured in 10% fetal bovine serum (HyClone, Logan, U.S.A.) and 1% PS (100 units/ml penicillin and 100 mg/ml streptomycin) medium with GlutaMAX (DMEM, Gibco, U.S.A.).

For d-glucose treatment, ARPE-19 cells were habitually passaged every 3–4 days, incubated in an incubator with 5% CO_2_ at 37°C. After ARPE-19 cells were grown to 80% confluence, the cells were plated at 1.5 × 10^4^ cells/well in six-well plates and treated with different concentrations of d-glucose (0, 5, 10, 20, 30, 50, 100 mM; Sigma–Aldrich, U.S.A.) for 24 h. For untreated ARPE-19 cells were added with 5.5 mM glucose into medium incubated at 37°C.

The day before cell transfection, ARPE-19 cells were seeded in a six-well plate at the concentration of 2 × 10^4^ cells per well and cultured in an incubator at 37°C with CO_2_. Then, the above cells were transfected with miR-200a-3p mimic (mimic), pcDNA-3.1-TGF-β2 (TGF-β2), or TGF-β2+ miR-200a-3p mimic (TGF-β2+mimic) with 30 μl Lipofectamine 3000 reagent (Invitrogen Life Technologies, U.S.A.) according to the manufacturer’s protocols. Cells without any treatment were recognized as the control group (NC). These cells were initially transfected for 6 h, then the culture medium was replaced with DMEM/f-12 medium containing 30 mM HG. After incubation for 24 h, the cells were subjected to the following experiments.

### Reverse transcription-quantitative polymerase chain reaction

The total RNA of clinical specimens and cell lines was extracted by using TRIzol reagent (QIAGEN, Germany) according to the manufacturer’s instructions. For mRNA detection, RNA samples were revere-transcribed into cDNA using the PrimeScript™ RT reagent kit with gDNA Eraser (TaKaRa, Japan). And reverse transcription (RT)-quantitative polymerase chain reaction (RT-qPCR) was performed with GoTaq qPCR Master Mix (TaKaRa, Japan) using the CFX96 Sequence Detection System (Bio-Rad, U.S.A.). For miRNA detection, RNA samples were reverse transcribed using the Mir-X miRNA First-Strand Synthesis Kit (TaKaRa, Japan) using Bio-systems 7300 Real-Time PCR systems. The sequence of the involved primers was designed and listed in Table 1. U6 and β-actin as an endogenous reference for miRNA and mRNA, respectively. RT-qPCR was performed in triplicate.

**Table 1:** Names and sequences of the primers

Name	Primer sequences
miR-200a-3p	F: 5′-CGTAACACTGTCTGGTAACGATGT-3′
	R: 5′-TGGTGTCGTGGAGTCG-3′
TGF-β2	F: 5′-TCCATCTGTGAGAAGCCACA-3′
	R: 5′-GGGTCATGGCAAACTGTCTC-3′
TNF-α	F: 5′-AGCCCCCAGTCTGTATCCTT-3′
	R: 5′-CTCCCTTTGCAGAACTCAGG-3′
IL-6	F: 5′-GCCCAAACACCAAGTCAAGT-3′
	R: 5′-TATAGGAAACAGCGGGTTGG-3′
IL-1β	F: 5′-CAGAAGTACCTGAGCTCGCC-3′
	R: 5′-AGATTCGTAGCTGGATGCCG-3′
MMP2	F: 5′-GATTGACGCTGTGTATGAGGC-3′
	R: 5′-TTCCAGGAGTCTGCGATGAG-3′
MMP9	F: 5′-GGCAACGGAGAAGGCAAAC-3′
	R: 5′-CCACTCGGGTAGGGCAGAA-3′
β-actin	F: 5′-CGTGCGTGACATCAAAGAGAAG-3′
	R: 5′-CCAAGAAGG AAGGCTGGA AAA-3′
U6	F: 5′-GCTTCGGCAGCACATATACTAAAAT-3′
	R: 5′-CGCTTCACGAATTTGCGTGTCAT-3′

Abbreviations: F, forward primer; R, reverse primer.

### Cell proliferation assay

Cell proliferation was assessed every 24 h using MTT assay (Sigma, U.S.A.) to determine relative cell growth. Briefly, ARPE-19 cells transfected with miR-200a-3p mimic, pcDNA3.1-pcDNA-3.1-TGF-β2, TGF-β2+miR-200a-3p mimic or pretreatment with 10 nM SRI-011381 (SRI; TGF-β2/Smad pathway activator, MedChem Express, Monmouth Junction, U.S.A.) were seeded in 96-well plates and then exposed to HG treatment. Then, 10 μl MTT (5 mg/ml) solution per well was added and cultured at 37°C and 5% CO_2_ for 4 h. Subsequently, the supernatant was discarded and the wells washed by PBS. Each well was added with 150 μl DMSO (Sigma, U.S.A.), and the MTT formazan crystals were dissolved. The absorbance was measured at 490 nm using a microplate reader (Bio-Tek, Winooski, U.S.A.).

### Flow cytometry

The Annexin V-FITC/Propidium Iodide (PI) apoptosis detection kit (Thermo Fisher, U.S.A.) was employed to determine cell apoptosis ratio according to the manufacturer’s instructions. Briefly, after transfection and HG treatment, 2 × 10^4^ cells were harvested, washed twice in PBS, and re-suspended in fixation fluid. And then, ARPE-19 cells were stained with 5 μl Annexin V-FITC and 2 μl PI for 15 min and incubated at room temperature. Cell apoptosis was then analyzed with flow cytometry using BD LSR II Flow Cytometry (BD Biosciences, U.S.A.).

### Dual-luciferase reporter gene assay

The PD-L1 fragment containing miR-200a-3p binding sites were synthesized to generate wild type (WT) or mutant type (MUT). The TGF-β2-WT and TGF-β2-MUT fragments were subcloned into the *Renilla* luciferase gene pGL3-Luciferase reporter vectors (Promega, U.S.A.) to generate pGL3-TGF-β2 (WT) and pGL3-TGF-β2 (MUT) vectors, respectively. After that, the above vectors were co-transfected with miR-200a-3p mimic or mimic control into HEK-293T cells for 24 h. Finally, the cells were lysed by using the Dual-luciferase assay kit (Promega, U.S.A.), and the luciferase activities were evaluated by the luminescence plate reader (Molecular Devices Inc, U.S.A.).

### Western blotting

The total proteins of the tissues and cells were extracted by using the RIPA lysis buffer purchased from Beyotime Biotechnology (Shanghai, China) according to the manufacturer’s protocol. The BCA kit (Beyotime Biotechnology, China) was used to quantify the protein concentration. After that, the target proteins were separated by conduction the electrophoresis with 10% SDS/polyacrylamide gel and transferred to the polyvinylidene fluoride (PVDF) membranes produced by Millipore (MA, U.S.A.). The 5% skim milk was diluted by TBS containing 0.1% Tween-20 and incubated with the membranes for 1 h at 37°C. The PVDF membranes were incubated overnight at 4°C with the primary rabbit antibodies against human TGF-β2 (1:1000, ab36495, Abcam, U.K.), Smad2 (1:2000, ab63576, Abcam, U.K.), Smad3 (1:2000, ab28379, Abcam, U.K.), Bax (1:1000, ab32503, Abcam, U.K.), Bcl-2 (1:1000, ab59348, Abcam, U.K.), cleaved caspase-3 (1:1000, ab2302, Abcam, U.K.), VEGF (1:1000, ab39638, Abcam, U.K.), MMP2 (1:1000, ab92536, Abcam, U.K.), MMP9 (1:1000, ab38898, Abcam, U.K.). The horseradish peroxidase-linked goat anti-rabbit IgG (1:2000, ab205718, Abcam, U.S.A.) was incubated with the membranes for 1 h at room temperature. The ECL Western blotting detection kit (Bio-Rad, U.S.A.) was employed to detect the optical density of the protein bands to evaluate the expression levels of the proteins.

### Animal model

All animal experiments were approved by the Animal Ethics Committee of Yunnan No.2 Provincial People’s Hospital (approval number: 2019179), and animal work took place in the central animal facility of the medical faculty at the Yunnan No.2 Provincial People’s Hospital. A total of 40 male Wistar rats (6–8 weeks old) were randomly divided into four groups (10 rats per group) as follows: (1) normal control group, all rats received an identical volume of citrate buffer solution. (2) DR model group, all rats received an intraperitoneal injection of streptozotocin (STZ; 60 mg/kg, Sigma, U.S.A.) which was dissolved in freshly prepared sodium citrate buffer (0.1 mM, pH 4.5). (3) DR+miR-200a-3p group, given agomir (miR-200a-3p; 3 mg/kg, Ribobio, China) through tail intravenous injection and then received an intraperitoneal injection of STZ. (4) DR+miR-200a-3p+SRI group, all rats were given SRI (TGF-β2/Smad pathway activator, 1 mg/kg) through tail intravenous injection and then received an intraperitoneal injection of STZ. Rats with blood glucose levels above 16.67 mM after STZ administration for 72 h were diagnosed with DR and used for further experiments. After 10 weeks of incubation, all rats were anesthetized with 300 mg/kg chloral hydrate and killed by dislocation and collected the retinal tissues for further experiments. Immunohistochemical staining was applied to observe the retinal vascular changes and examine the expression of VEGF according to the previous studies [22].

### Enzyme-linked immune sorbent assay

The expression levels of IL-6, IL-1β, and TNF-α in the retina of rats were detected according to the method of enzyme-linked immune sorbent assay (ELISA) kit (R&D Systems, U.S.A.).

### Statistical analysis

All data were collected and presented as the mean ± standard deviation. The data were then analyzed using SPSS 22.0 software (IBM, U.S.A.). Student’s *t* test and one-way ANOVA were used to evaluate statistical significance. *P*<0.05 was considered to indicate a statistically significant difference.

## Results

### Effect of HG on the cell viability, apoptosis, and inflammation in ARPE-19 cells

**Figure 1:**
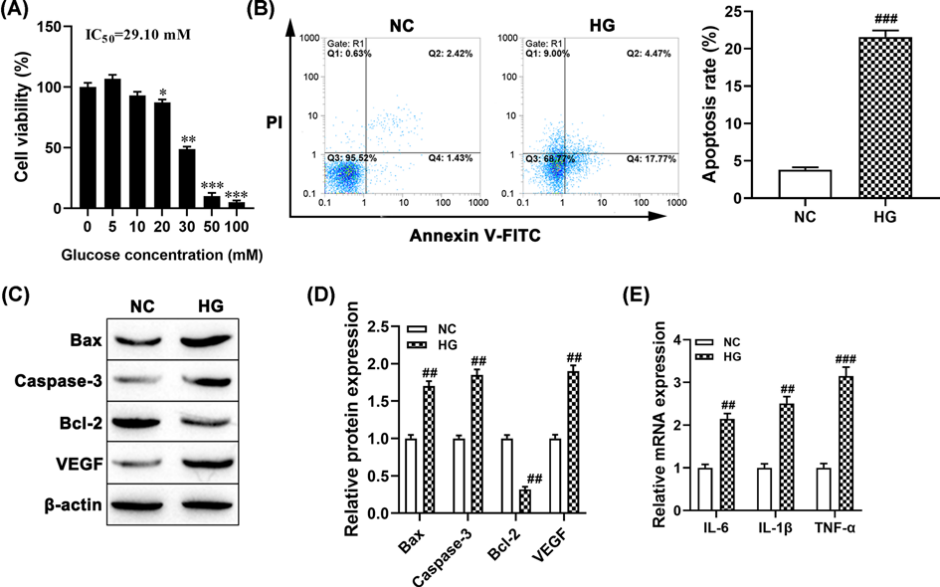
HG inhibited cell viability and induced cell apoptosis and inflammation in ARPE-19 cells (**A**) ARPE-19 cells were exposed to different concentrations of d-glucose (0–100 mM) treatment for 24 h, and untreated cells acted as a control, after which cell viability was evaluated by MTT; 30 mM was taken as HG concentration to expose ARPE-19 cells, *P<0.05, **P<0.01, ***P<0.001, compared with the untreated group. (**B**) Flow cytometry was used to examine cell apoptosis in ARPE-19 cell, ^###^*P*<0.001, compared with the NC group. (**C,D)** The protein levels of Bax, caspase-3, Bcl-2, and VEGF were detected using Western blotting. ^##^*P*<0.01, compared with the NC group. (**E**) The mRNA levels of inflammatory cytokines were measured using RT-qPCR, ^##^*P*<0.01, ^###^*P*<0.001, compared with the NC group.

Previous studies suggested that HG was used to trigger the DR model in ARPE-19 cells, the effect of different concentrations of HG on cell viability, apoptosis, and inflammation was examined. Primarily, MTT assay results showed that ARPE-19 cells treated with different concentrations of d-glucose for 24 h significantly inhibited cell viability (Figure 1A). The IC_50_ value of glucose was calculated as 29.10 and 30 mM glucose, acts as HG, was selected to induce ARPE-19 cell injury for further experiments. Meanwhile, the rate of apoptotic cells of ARPE-19 treated with HG was higher than that of the control group (*P*<0.001, Figure 1B). Similarly, Western blotting showed that HG treatment significantly promoted the expression levels of pro-apoptotic protein Bax and Caspase-3 (both *P*<0.01, Figure 1C,D), but decreased anti-apoptotic protein Bcl-2 expression (*P*<0.01, Figure 1C,D). In addition, the expression of VEGF protein was markedly enhanced in HG pretreated ARPE-19 cells compared with the control group (*P*<0.01, Figure 1C,D). Furthermore, we observed that the expression levels of IL-6, IL-1β and TNF-α, were higher in HG-induced ARPE-19 cells than the NC group (all *P*<0.01, Figure 1E). These results indicated that the HG-induced ARPE-19 cell injury model was successful.

### Effect of miR-200a-3p on proliferation and apoptosis of HG pretreated ARPE-19 cells

**Figure 2:**
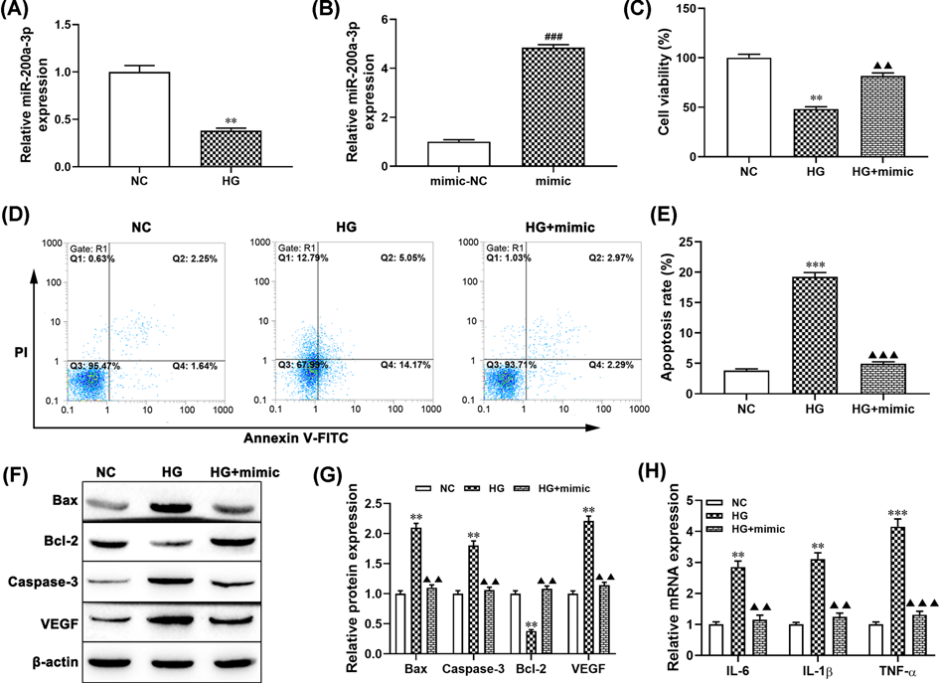
miR-200a-3p overexpression reduced HG-induced ARPE-19 cell injury ARPE-19 cells were treated with 30 mM HG for 24 h. (**A**) The expression of miR-200a-3p in ARPE-19 cells was determined by RT-qPCR. (**B**) RT-qPCR was performed to examine the expression of miR-200a-3p in cells transfected with miR-200a-3p mimic and NC-mimic, **P<0.01, compared with the NC group; ^###^*P*<0.001, compared with the mimic-NC group. (**C**) Cell proliferation was detected by MTT assay in ARPE-19 cells transfected with miR-200a-3p mimic and then incubated with 30 mM HG, **P<0.01, compared with the NC group; ^▲▲^*P*<0.01, compared with the HG group. (**D,E**) Cell apoptosis was determined by flow cytometry in ARPE-19 cells transfected with miR-200a-3p mimic and then incubated with 30 mM HG, ***P<0.001, compared with the NC group; ^▲▲▲^*P*<0.001, compared with the HG group. (**F,G**) The protein levels of Bax, caspase-3, Bcl-2, and VEGF in ARPE-19 cell treated with HG or HG plus miR-200a-3p mimic were detected using Western blotting, **P<0.01, compared with the NC group; ^▲▲^*P*<0.01, compared with the HG group. (**H**) The mRNA levels of IL-6, TNF-α and IL-1β were measured using RT-qPCR, **P<0.01, compared with the NC group; ***P<0.001, ^▲▲^*P*<0.01, ^▲▲▲^*P*<0.001, compared with the HG group.

Increasing evidence confirmed that miRNAs exerted an important role in the proliferation and apoptosis of ARPR-19 cells exposed to HG [23]. Herein, we examined the expression level of miR-200a-3p in ARPE-19 cells treated with HG. As shown in Figure 2A, the expression level of miR-200a-3p in ARPE-19 cells treated with HG significantly decreased compared with the NC group (*P*<0.01). Moreover, we further examined the gain-of-function of miR-200a-3p in the proliferation and apoptosis of ARPE-19 cells and transfected miR-200a-3p mimic into ARPE-19 cells (*P*<0.001, Figure 2B). MTT assay showed that overexpression of miR-200a-3p notably alleviated the inhibitory effect of HG on the proliferation of ARPE-19 cells (*P*<0.01, Figure 2C). Flow cytometry analysis results showed that up-regulation of miR-200a-3p significantly decreased the apoptosis rate of ARPE-19 cells treated with HG compared with the HG alone group (*P*<0.01, Figure 2D,E). Meanwhile, Western blotting revealed that the expression levels of Bax, Caspase-3, and VEGF were decreased in the miR-200a-3p+HG group compared with the HG treatment alone group (all *P*<0.01, Figure 2F,G), while enhancing the expression of Bcl-2 (*P*<0.01). Furthermore, the mRNA expression levels of IL-6, IL-1β, TNF-α in the miR-200a-3p+HG group were lower than the HG alone group (*P*<0.01, *P*<0.001, Figure 2H). These results strongly implied that overexpression of miR-200a-3p attenuated HG-induced cell apoptosis and inhibited cell viability in ARPE-19 cells.

### miR-200a-3p negatively regulated TGF-β2 expression

**Figure 3:**
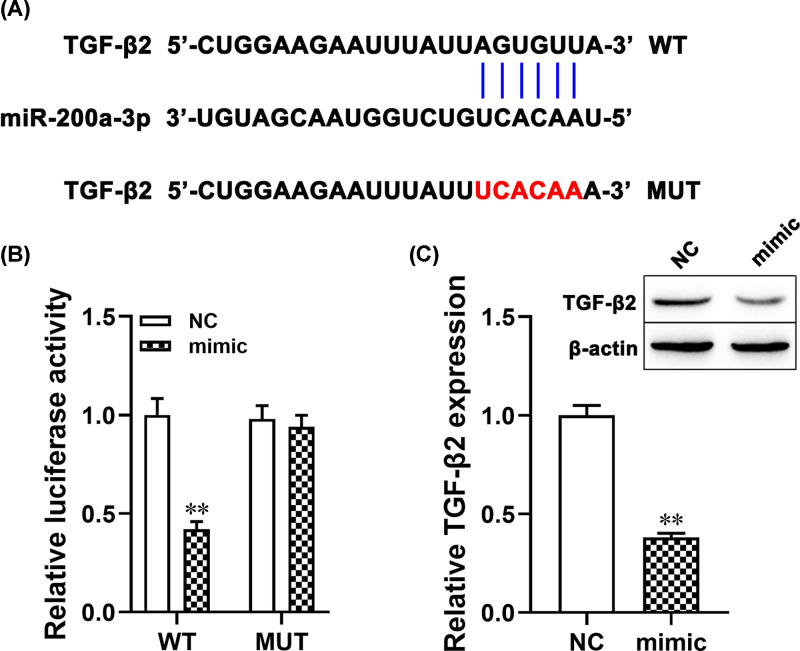
miR-200a-3p negatively regulated TGF-β2 expression (**A**) Bioinformatics analysis indicated the putative binding sites and corresponding mutant region for miR-200a-3p within TGF-β2. (**B**) Dual-luciferase reporter gene assay for WT and MUT TGF-β2 3′UTR luciferase vectors in HEK-293T cells transfected with control or miR-200a-3p mimic. (**C**) The expression of TGF-β2 protein in ARPE-19 cells transfected with miR-200a-3p mimic was determined by Western blotting. **P<0.01, compared with the NC group.

The miR-200a-3p target gene was obtained from the bioinformatics database (TargetScan; http://www.targetscan.org/vert_71/). The bioinformatics analysis results showed that miR-200a-3p had a binding site with TGF-β2, as shown in Figure 3A. To further investigate the relationship between miR-200a-3p and TGF-β2 was checked by the dual-luciferase reporter gene system. The result showed that the co-transfection of miR-200a-3p mimic and TGF-β2-WT significantly decreased the luciferase activity (*P*<0.01, Figure 3B), but co-transfection of miR-200a-3p mimic and TGF-β2-MUT did not change the luciferase activity. Moreover, Western blotting showed that overexpression of miR-200a-3p decreased the expression of TGF-β2 protein (*P*<0.01; Figure 3C). Taken together, these results suggested that TGF-β2 was the direct target of miR-200a-3p which negatively regulated TGF-β2 expressions.

### miR-200a-3p targets TGF-β2 to protect HG-induced ARPE-19 cells injury *in vitro*

**Figure 4:**
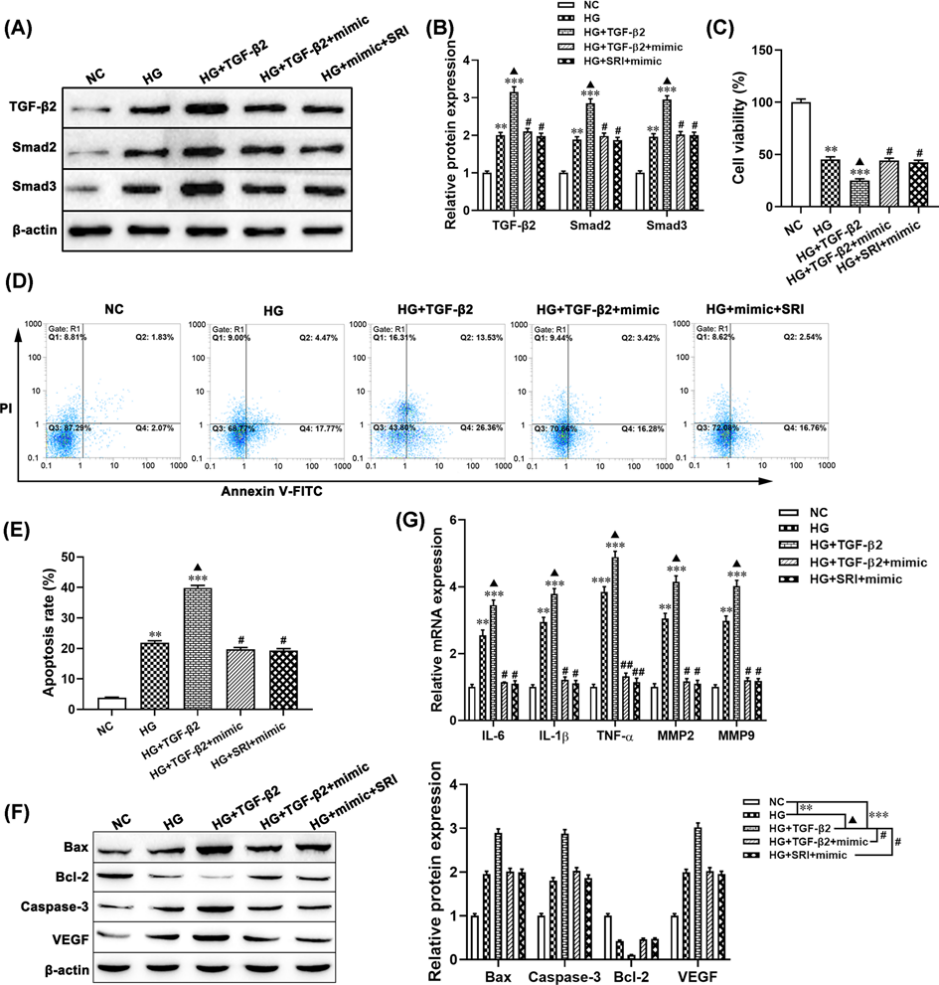
miR-200a-3p targets TGF-β2 to protect HG-induced ARPE-19 cell injury *in vitro* ARPE-19 cells were treated with HG (30 mM), HG (30 mM)+pcDNA3.1-TGF-β2, HG (30 mM)+pcDNA3.1 TGF-β2+miR-200a-3p mimic, HG (30 mM)+SRI (5 mM)+miR-200a-3p mimic. (**A,B**) The protein levels of TGF-β2, Smad2, and Smad3 were detected using Western blotting. (**C**) MTT assay was used to measure cell viability. (**D,E**) Flow cytometry was performed to examine cell apoptosis. (**F**) Western blotting was applied to detect the protein levels of Bax, caspase-3, Bcl-2, and VEGF. (**G**) The mRNA levels of IL-6, IL-1β, and TNF-α were measured using RT-qPCR. **P<0.01, ***P<0.001, compared with the NC group; ^▲^*P*<0.05, compared with the HG group; ^#^*P*<0.05, ^##^*P*<0.01, compared with the HG+TGF-β2 group. Abbreviation: SRI, TGF-β2/Smad pathway activator SRI-011381.

To further determine whether miR-200a-3p regulates HG-induced cell injury though targeting TGF-β2 in ARPE-19 cells. Western blotting results showed that overexpression of TGF-β2 enhanced the expression of TGF-β2, Smad2 and Smad3 in HG treated ARPE-19 cells compared with the NC group or HG alone group (Figure 4A,B), while no significant difference between the HG+TGF-β2+miR-200a-3p mimic group or HG+miR-200a-3p mimic+SRI group and HG treatment alone group. MTT analysis results showed that overexpression of TGF-β2 markedly decreased cell viability of HG pretreated ARPE-19 cells compared with the only HG treatment group (*P*<0.05, Figure 4C). Flow cytometry analysis results showed that up-regulation of TGF-β2 increased the percentage of apoptotic ARPE-19 cells treated with HG (*P*<0.05, Figure 4D,E). Moreover, the apoptosis-related proteins Bax, Bcl-2, and Caspase-3 detected by Western blotting were consistent with the result of the flow cytometry analysis (Figure 4F). Furthermore, the VEGF, MMP-2, MMP-9, TNF-α, IL-6, and IL-1β levels in ARPE-19 cells by transfecting with pcDNA-TGF-β2 plus HG were significantly increased compared with the only HG treated group (all *P*<0.05, Figure 4G). However, co-transferred with miR-200a-3p mimic+SRI or miR-200a-3p mimic+pcDNA-TGF-β2 in HG pretreated ARPE-19 cells abolished the effect of TGF-β2 up-regulation on the cell viability and the inflammatory cytokines mRNA expression (Figure 4). These results suggested that up-regulated miR-200a-3p alleviated HG-induced ARPE-19 cell injury by blocking the TGF-β2/Smad pathway.

### Effect of miR-200a-3p on the progression of DR through regulating TGF-β2/Smad pathway *in vivo*

**Figure 5:**
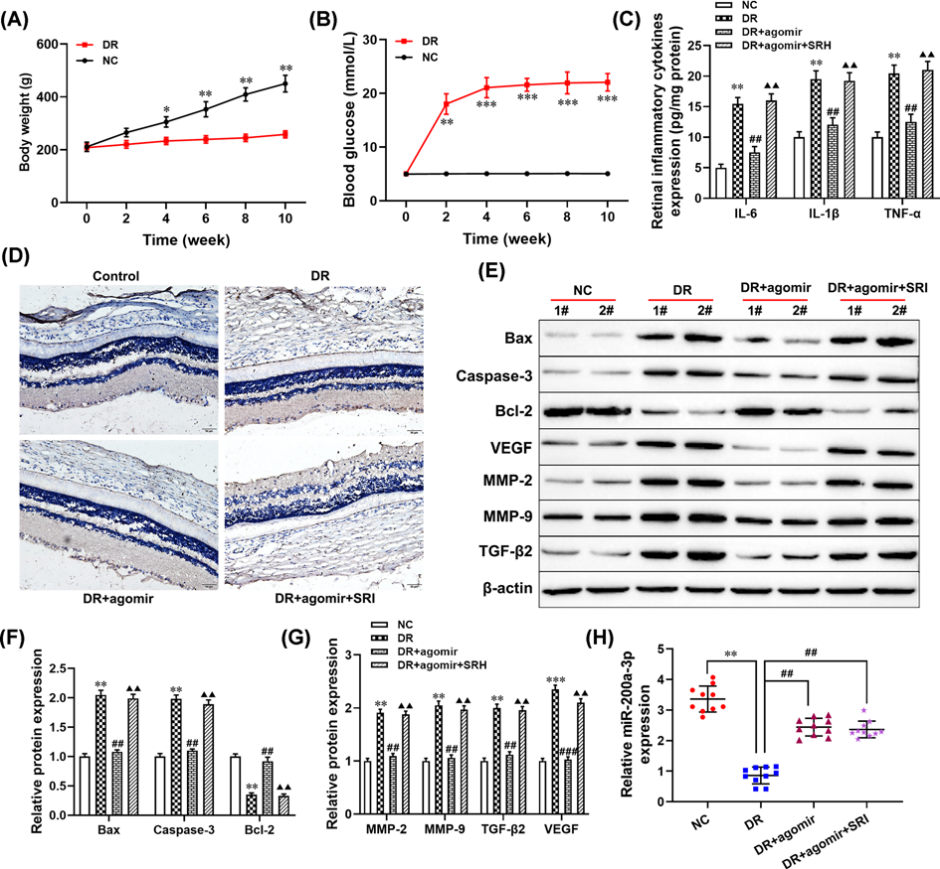
Overexpression of miR-200a-3p attenuated the progression of DR through regulating the TGF-β2/Smad pathway *in vivo* DR model group, all rats received an intraperitoneal injection of STZ (60 mg/kg) which was dissolved in freshly prepared sodium citrate buffer (0.1 mM, pH 4.5); DR+miR-200a-3p group, given agomir (miR-200a-3p; 3 mg/kg) through tail intravenous injection and then received an intraperitoneal injection of STZ; DR+miR-200a-3p+SRI group, all rats were given SRI (1 mg/kg) through tail intravenous injection and then received an intraperitoneal injection of STZ. (**A**) The body weight of all mice was measured, *P<0.05, **P<0.01, compared with the NC group. (**B**) The level of blood glucose in all mice was examined, **P<0.01, ***P<0.001, compared with the NC group. (**C**) The levels of inflammatory cytokines were detected using ELISA. (**D**) Immunohistochemical staining was performed to detect the expression of VEGF. (**E**–**G**) The protein levels of Bax, caspase-3, bcl-2, MMP-2, MMP-9, TGF-β2, and VEGF were measured using Western blotting. (**H**) RT-qPCR was used to detect the expression of miR-200a-3p. **P<0.01, compared with the NC group; ^##^*P*<0.01, compared with the DR group; ^▲▲^*P*<0.01, compared with the HG+agomir group.

To further examine whether miR-200a-3p-elevated alleviated the progression of DR by blocking the TGF-β2/Smad pathway in the DR rats’ model. First, the body weight and the levels of blood glucose in DR rats from normal and STZ-induced diabetic rats at 0, 2, 4, 6, 8, and 10 weeks were measured to evaluate whether the construction of the diabetic rats’ model was successful or not. There was significant weight loss and blood glucose increased in DR rats compared with the age-matched control group (NC; Figure 5A,B). Next, immunohistochemical staining demonstrated that the positive expression of VEGF in the retina tissues of the DR model was higher than that of the NC group, as well as, overexpression of miR-200a-3p significantly decreased VEGF positive expression in retinal tissues (Figure 5D). However, activation of TGF-β2/Smad pathway (SRI-011381, SRI) markedly down-regulated the inhibitory effect of miR-200a-3p on VEGF expression in DR rats (Figure 5D). Moreover, ELISA results showed that the levels of inflammatory cytokines secreted in the DR+agomir group were lower than that in the DR group (all *P*<0.01, Figure 5C), but no significant difference was found between the DR+agomir+SRI group and DR group. Western blotting analysis results revealed that the expression levels of apoptotic-related proteins, TGF-β2, VEGF, MMP-2, and MMP-9 in retinal tissues of the DR model were significantly overexpressed compared with the NC group (all *P*<0.01, Figure 5E–G), while overexpression of miR-200a-3p significantly repressed these effects. However, inhibition of the TGF-β2/Smad pathway alleviated the protection effect of miR-200a-3p (Figure 5E–G). Furthermore, RT-qPCR results showed that the expression of miR-200a-3p was down-regulated in the retinal tissues of the DR model compared with the NC group (*P*<0.001, Figure 5H), but overexpressed in the DR+agomir group (*P*<0.01). Taken together, these results collectively illustrated that overexpression of miR-200a-3p alleviated the progression of DR via blocking the TGF-β2/Smad pathway.

## Discussion

As the incidence of DR is increasing all over the world, it is necessary to find the regulatory mechanism or biomarker in the occurrence and development of DR for clinical treatment and diagnosis. In the present study, we found that miR-200a-3p was down-regulated in ARPE-19 cells treated with HG and the retina tissues of DR rats, and overexpression of miR-200a-3p promoted cell viability and decreased the expression levels of MMP-2, MMP-9, TNF-α, IL-1β, and VEGF, which was reversed by up-regulating TGF-β2. The results illustrated that elevated miR-200a-3p alleviated HG-induced cell apoptosis and immunoreaction through down-regulating TGF-β2 in DR progression.

Previous studies found that activation of the TGF-β2/Smad pathway contributed to the progression of DR [24]. TGF-β2 could modulate the regulation of cell differentiation, proliferation, apoptosis, epithelial–mesenchymal transition (EMT), and immune response through regulating its downstream Smad family proteins, and eventually promoted the development of DR [18,25]. Yang et al. proved that the TGF-β2/Smad pathway plays an important role in the proliferation, migration, and EMT of HG pretreated human lens epithelial cells [26]. Our studies confirmed that overexpression of TGF-β2 promoted HG-induced cell apoptosis, proinflammatory cytokines secretion, and VEGF expression in ARPE-19 cells. In addition, many studies have elucidated TGF-β receptor-induced responses were mediated through multiple signaling pathways, including ERK/p38/JNK [27], PI3K/Akt/mTOR [28], and NF-κB [29]. In the present study, our data confirmed that HG-induced cell apoptosis and VEGF elevated in ARPE-19 cells via activating the TGF-β2/Smad pathway. Moreover, the mechanism of TGF-β2 modulated migration and EMT of vascular endothelial cells to regulate DR progression will be investigated in our further study.

More and more studies reported that miR-200 families were found to be aberrantly expressed in malignant tumors [30], DR, and pulmonary fibrosis [31]. Herein, our results demonstrated that miR-200a-3p expression was decreased in HG pretreated ARPE-19 cells and retinal tissues of DR rats. Such as, Lo et al. reported that miR-200a-3p was lower expressed in retinal tissues of DR rats [32]. Recently, some miRNAs mediated the progression of DR by regulating the proliferation, apoptosis, inflammatory damage, and MMPs secretion of retinal pigment epithelial (RPE) cells. For example, Ding et al. found that miR-200b/c up-regulation suppressed the proliferation and migration of RPE cells [33]. Repression miR-34a could decrease HG-induced apoptosis and inflammation cytokines secretion of RPE cells treated with HG [32]. MMP-2 was significantly decreased in RPE cells through up-regulating miR-29a [34]. In addition, the miR-200 family mediated the development and progression of many diseases through regulating TGF-β [35,36]. For example, miR-200a [37], miR-200b [38], miR-200c [39]. Importantly, our results found that overexpression of miR-200a-3p targets TGF-β2 to suppress HG-induced ARPE-19 cells apoptosis and inflammation cytokines secretion *in vitro*.

In conclusion, our results confirmed that increased miR-200a-3p suppressed HG-induced cell apoptosis and immunoreaction by blocking the TGF-β2/Smad pathway in DR progression. Furthermore, this mechanism may provide new biomarkers and targets for the diagnosis and treatment of DR.

## Data Availability

The datasets used and/or analyzed in the current study are available from the corresponding author on request.

## Abbreviations

Bax: B-cell lymphoma protein-2-associated X
Bcl-2: B-cell lymphoma protein 2
DR: diabetic retinopathy
ELISA: enzyme-linked immune sorbent assay
EMT: epithelial–mesenchymal transition
HG: high glucose
IL: interleukin
miRNA: microRNA
MMP: matrix metalloprotease
MTT: 3-(4,5-dimethylthiazol-2-yl)-2,5-diphenyl-trtrazolium bromide
MUT: mutant type
PI: propidium iodide
PVDF: polyvinylidene fluoride
RPE: retinal pigment epithelial
RT-qPCR: reverse transcription-quantitative polymerase chain reaction
Smad: sterile alpha motif domain containing
STZ: streptozotocin
TGF-β: transforming growth factor-β
TNF-α: tumor necrosis factor alpha
VEGF: vascular endothelial growth factor
WT: wild type
